# Chronic neuropathic ulcer is not the most common antecedent of lower limb infection or amputation among diabetics admitted to a regional hospital in Jamaica: results from a prospective cohort study

**DOI:** 10.1186/s12893-015-0091-4

**Published:** 2015-09-21

**Authors:** Jeffrey M. East, Delroy A. Fray, Dwayne E. Hall, Chapman A. Longmore

**Affiliations:** Department of Surgery, Cornwall Regional Hospital (CRH), Montego Bay, Jamaica; Department of Surgery, Radiology, Anaesthesia and Intensive Care, University of the West Indies, Mona, Kingston 6 Jamaica

**Keywords:** Soft tissue infection diabetes, Abscess diabetes, Diabetic foot ulcer, Trauma diabetes, Puncture wounds foot, Critical limb ischemia diabetes, Diabetes Caribbean, Diabetes developing countries

## Abstract

**Background:**

Guidelines of the International Consensus on the Diabetic Foot state that “Amputation of the lower extremity or part of it is usually preceded by a foot ulcer”. The authors’ impression has been that this statement might not be applicable among patients treated in our institution. A prospective cohort study was designed to determine the frequency distribution of antecedents of lower limb infection or gangrene and amputation among adult diabetics admitted to a Regional Hospital in western Jamaica.

**Methods:**

Adult diabetics admitted to Hospital with a primary diagnosis of lower limb infection and/or gangrene were eligible for recruitment for a target sample size of 126. Thirty five variables were assessed for each patient-episode of infection and/or gangrene, main outcome variable being amputation during admission or 6-months follow-up. Primary statistical output is the frequency distribution of antecedents/precipitants of lower limb infection and/or gangrene. The data is interrogated by univariate and multivariable logistic regression for variables statistically associated with the main antecedent/precipitant events.

**Results:**

Data for 128 patient-episodes were recorded. Most common antecedents/precipitants, in order of decreasing frequency, were idiopathic acute soft tissue infection/ulceration (30.5 %, CI; 22.6–39.2 %), chronic neuropathic ulcer (23.4 %, CI; 16.4–31.7 %), closed puncture wounds (19.5 %, CI; 13.1–27.5 %) and critical limb ischemia (7.8 %, CI; 3.8–13.9 %). Variables positively associated with non-traumatic antecedents/precipitants at the 5 % level of significance were male gender and non-ulcerative foot deformity for idiopathic acute soft tissue infection/ulcer; diabetes >5 years, previous infection either limb, insulin dependence and peripheral sensory neuropathy for chronic neuropathic ulcer and older age, diabetes >5 years, hypertension, non-palpable distal pulses and ankle-brachial index ≤0.4 for critical limb ischemia.

**Conclusions:**

Chronic neuropathic ulcer accounted for only 23.4 % of lower limb infections and 27.7 % of amputations in this population of diabetics, making it the second most common antecedent of either after acute idiopathic soft tissue infection/ulcer at 30.5 and 34.7 % respectively. Trauma as a group (defined as closed puncture wounds, lacerations, contusion/blunt trauma and burns) also accounted for a greater number of lower limb infections but fewer amputations than chronic neuropathic ulcer, at 32 and 19.5 % respectively.

## Background

Peña has issued a call for implementation of the guidelines of the International Consensus on the Diabetic Foot in the Caribbean with the hope of staunching current and predicted rates of lower limb complications and amputation among diabetics in this population [1], reportedly already very high [2, 3]. The Consensus guidelines recommend a comprehensive program of lifestyle changes, patient, community and healthcare provider education, early diagnosis and timely medical and surgical intervention and include a detailed, practical manual on management and prevention of the diabetic foot [4].

There are several factors militating against the potential effectiveness of any lower limb amputation reduction strategy among diabetics in Jamaica, were such a strategy to be implemented at this time. Poverty, currently at high levels in Jamaica, increases the risk of developing diabetes in the first place [5]. Poverty also decreases the ability of the sufferer to control the disease, given the relatively expensive medication and diet required, impairs maintenance of living conditions conducive to protection of the feet, such as proper footwear and an environment free of potentially injurious implements, and increases the risk of amputation among diabetics [6]. There is an almost complete absence of primary specialist foot care services in the extensive public hospital and primary care network. Access to consultant level General Surgery and Orthopaedic care during the earliest stages of lower limb infections, when surgical intervention would be most beneficial, is difficult. It is not until infections become so severe that limb and life are threatened that patients are able to access this level of care.

Low general and diabetes-specific education compound the difficulty of achieving acceptable levels of diabetes control and foot self-care and protection [7, 8]. There is evidence indicating that patients resist compliance with treatment and preventive interventions if they do not understand their disease and the rationale for intervention [9], particularly as viewed from the perspective of deep-seated cultural beliefs [10]. The authors are not aware of any comprehensive interventions, at the level of the individual diabetic in Jamaica, to explain the nature of the disease and the rationale for treatment and prevention measures in language that can be understood by laypersons.

In addition to these deficiencies of social and health care infrastructure, there are inadequate data on the profile and frequency distribution of conditions preceding and precipitating lower limb infections and gangrene among the local diabetic population. This information will be critical for planning appropriately targeted infection and amputation reduction interventions for optimum effectiveness. Wright-Pascoe et al. [11] presented a retrospective review of 44 patients treated for diabetic foot ulcers at the University Hospital of the West Indies in Kingston. It is unclear how many of these had active infections as opposed to non-invasive contamination. Thirty one patients (70.6 %) had a history of prior (presumably chronic) ulceration and 29.4 % had a history of trauma (11.8 % puncture wounds). Review of the literature failed to reveal any other research publications on the antecedents and other determinants of lower limb infections and gangrene among diabetics in Jamaica.

The introductory section of the Consensus guidelines [4] states that “Amputation of the lower extremity or part of it is usually preceded by a foot ulcer”. It is presumed that by “foot ulcer” the authors mean chronic ulceration and neither the acute ulceration which follows breakdown of the skin overlying acute soft tissue infections nor acute iatrogenic wounds resulting from surgical debridement of closed soft tissue infections and puncture wounds. “Usually” is taken to mean either that a majority (> 50 %) of amputations are preceded by “foot ulcer” or “foot ulcer” is a more common antecedent of amputation than any other event. Only one paragraph of the guidelines is dedicated to the overall strategy for treating non-ulcerative pathology with the main focus of the document being prevention and treatment of chronic ulcers. Similar positions are taken in guidelines of the American College of Foot and Ankle Surgeons [12], the American Diabetes Association [13] and the Infectious Diseases Society of America (IDSA) [14]. Some publications are even more specific, stating that 85 % of lower limb amputations in diabetics are preceded by ulceration (implying chronic, neuropathic ulceration) [15, 16].

It has been our impression at the Cornwall Regional Hospital (CRH) in western Jamaica that lower limb amputations in diabetics are almost always preceded by severe infection or gangrene, that such infections are caused by a multiplicity of foot lesions and that possibly a majority (> 50 %) of lower limb infections and gangrene in diabetics are not preceded by chronic ulceration. If this impression is correct, a surveillance, prevention and early intervention strategy based on the presumption that most diabetic foot infections and amputations are preceded by chronic ulceration is unlikely to be maximally effective in this population.

This study is a prospective observational design aimed primarily at identifying the frequency distribution of proximate antecedent and precipitating events for lower limb infections and gangrene among adult diabetics admitted to the CRH with either condition as the primary diagnosis. The frequency distribution of amputation by antecedent event is also determined and the data is interrogated to identify patient-specific variables that might be associated with the odds of presenting with a given antecedent event. It is expected that the study will inform a targeted strategy, via prevention, early detection and treatment of identified antecedents, for prevention and reduction of diabetic lower limb infections and gangrene and ultimately amputations in the population served by the CRH.

## Methods

The research protocol was approved by the separate ethics committees of the Western Regional Health Authority, St. James, Jamaica, and of the University of the West Indies at Mona, Kingston, Jamaica. Informed consent was received from all participants.

Data were collected exclusively at the CRH, a 400 bed public referral hospital in Montego Bay, St. James, Jamaica. All patients 18 years old and over with diabetes mellitus admitted to the institution with a primary diagnosis of bacterial infection and/or gangrene of a lower limb were eligible for inclusion. Infection is defined as purulence or two or more other local signs of inflammation [17] in any tissue or part of the lower limb and gangrene is defined as the presence of necrosis. These criteria were meant to capture only those patients with infection of sufficient severity and acuity to pose an imminent threat to the limb and exclude those with merely contaminated chronic ulcers, who would not usually be admitted. Patients with impaired cognition persisting for the duration of the admission period were excluded.

Patients were to be recruited consecutively until the target sample size was achieved. Target sample size calculation was based on the expected proportion of patients with a traumatic incident as the antecedent event. Wright-Pascoe et al. [11] retrospectively identified trauma as being associated with the diabetic foot in 29.4 % of 44 patients in Kingston, Jamaica, but “trauma” was not defined and “diabetic foot” apparently included a mix of patients with active infections and indolent ulcers, the latter group not being the primary focus of this study. Sample size calculation was based on the prevalence of trauma in that study, despite the difference in target population from this study. To detect a stable risk of a traumatic event (here defined as blunt injury, puncture wounds, lacerations and burns), as the precipitant for lower limb infection, of 30 % with a 95 % confidence interval of 22 % to 38 %, the number of patients needed is 126 [18].

Islam et al. [19] identified trauma (not defined) as the precipitating event in 50.7 % (54.5 %, with addition of lacerations and burns) of 446 diabetic patients with foot infections assessed prospectively in Trinidad and Tobago. This may more closely reflect the proportion of patients with trauma as the antecedent of lower limb infection in our study population and could be a more reasonable basis for the sample size calculation. Sample size needed to detect a risk of trauma of 50 % with a confidence interval of 40 to 60 % is 96 [18]. Target sample size for this study was therefore set at the higher number of 126 derived from the prior calculation, in the interest of achieving a model with greater power.

Data, based on a combination of interview, examination and investigation, were recorded unto a pre-coded form. The unit for each case record is a patient-episode, defined as hospital admission for infection or gangrene in one lower limb and including all subsequent admissions related to that same event in that same limb over the subsequent 6 months follow-up period. Admissions for re-infection related to the same event and limb occurring beyond 6 months after the index admission were recorded as separate patient-episodes, as were infections or gangrene in the other lower limb requiring admission at any time during the recruitment period. Bilateral infections were therefore treated as two, separate patient-episodes.

Variables extracted from the prospectively recorded database for each patient-episode for the purpose of this study are displayed in Table 1. Those data items that could be so verified were checked for accuracy and consistency against hospital records. Data were entered into a STATA Version 11 database for statistical analysis.

**Table 1:** Variables extracted and their frequency distribution among 128 patient-episodes of lower limb infection and/or gangrene

Demographic, behavioural & historical variables		Clinical, test and outcome variables	
Age – mean (range) in years)	58.4 (28–94)	Blood glucose on admission – mean (range) in mmol/L	20.4 (5.6–42)
Sex - no.(%) female)	82 (64.1 %)	Insulin dependent – yes (%)	82 (64.1 %)
^a^Social class IV & V - no.(%)	113 (88.3 %)	^e^Major comorbidities – yes (%)	95 (74.2 %)
How long diabetic – mode (range) in years)	10–20 (0– < 30)	Time to first medical attention – mean (range) in days	7 (1–42)
^b^Strenuous activity - no.(%)	60 (46.9 %)	Time to admission – mean (range) in days	13 (1–120)
First degree relatives with diabetes – yes (%)	99 (77.3 %)	Lower limb affected – right (%)	69 (53.9 %)
Current smoker (or stopped <6 months) – yes (%)	19 (14.8 %)	Nature of affliction - infection vs gangrene (%)	124 (96.9 %)
Ever smoked (stopped >6 months) – yes (%)	57 (47.5 %)	Part of limb infection started – forefoot (%)	97 (75.8 %)
^c^Current alcohol intake – none (%)/moderate (%)	101 (78.9 %) 26 (20.3 %)	Presence of chronic ulcer – yes (%)	42 (32.8 %)
Previous infections affected limb – yes (%)	45 (37.5 %)	^f^Non-ulcerative foot deformity – yes (%)	28/86 (32.6 %)
Previous infections either limb – yes (%)	65 (50.8 %)	^g^Osteomyelitis – yes (%)	19/83 (22.9 %)
Part of foot affected (if trauma) – forefoot (%)	33/41 (80.5 %)	Any distal pulse not palpable – yes (%)	34 (26.6 %)
Implement causing injury (if trauma) (see text)		Ankle-brachial index - no. < 0.9 (%)	42 (32.8 %)
Event preceding a chronic ulcer (see text)		^h^Peripheral sensory neuropathy – yes (%)	108 (84.4 %)
Ever instructed in foot care – yes (%)	99 (77.3 %)	^i^Event preceding infection/gangrene (see Table 2)	
Diabetic foot care behaviour – appropriate (%)	81 (63.3 %)	^j^Amputation – no. (%)	75/124 (60.5 %)
^d^Foot care by professional – yes (%)	17 (13.3 %)	^k^Major amputation – no. (%)	41/124 (33.1 %)

^a^According to the 1990 United Kingdom Registrar General’s social classification [28]

^b^Any regular physical activity, occupational or recreational, resulting in excessive or abnormal stresses to the feet, such as jogging, prolonged walking (>1 mile) or heavy physical labour

^c^Only one patient admitted to heavy alcohol intake

^d^Foot care professionals include podiatrists, foot nurses and pedicurists

^e^Includes hypertension, renal failure, heart disease, etc

^f^Any non-ulcerative abnormality interfering with the normal contour of the foot, such as bony deformity, arch distortion or callus. Patients with chronic ulcer were not assessed for other contour abnormalities

^g^Patients who obviously required amputation were not assessed for osteomyelitis

^h^As tested by the 10 g monofilament test

^i^As determined by research assistants (surgical residents) and verified by the principal investigator. The distribution of this variable is the main focus of this study

^j^Amputation at any level occurring during index admission or within 6 months of it. In four patient-episodes, patients were lost to follow-up after discharge from hospital

^k^Amputation proximal to the toes (transmetatarsal, below knee or above knee)

The primary output of this study is the frequency distribution of events or conditions leading up to or precipitating serious lower limb infection and/or gangrene and amputation in this sample of diabetics. The data is then interrogated, by univariate testing and multivariable regression, for statistically significant associations between those antecedent events occurring at a high frequency and all other potential confounding or modifying variables.

## Results

Between 16th March, 2011 and 25th March, 2013, data were collected for 128 patient-episodes of lower limb infection and/or gangrene among 124 diabetic patients from 150 admissions. However, hospital medical records statistics revealed that there had been 393 admissions of this type of case over that period, meaning that 243 admissions were missed by research assistants collecting prospective data. This admission rate (194/year) makes lower limb infection and/or gangrene in diabetics the 3rd leading cause of admission to General Surgery wards of this regional hospital, following trauma (gunshot wounds, stab wounds, motor vehicle accidents, industrial accidents, etc.) at 302/year and cancer at 240/year.

Unrecorded patient-episodes appeared to be missing completely at random (MCAR), seeming more likely to occur when the surgical service was particularly busy and residents had less time available for data collection. Given that most variables collected for this study cannot be verified or accurately measured retrospectively from hospital patient notes, it is not possible to conclusively test the assumption of MCAR by way of a comprehensive comparison of missed and study samples. Nevertheless, some key variables were extracted from medical records for a randomly selected sample of 125/194 missed patients (128 patient-episodes) for the purpose of comparison with study data. This comparison showed that study data did not differ from missed sample data by age (*P* = 0.07, *t*-test), sex (*P* = 0.25, chi-squared test), affected limb (*P* = 0.8, chi-squared test), dry gangrene versus infection/wet gangrene (*P* = 0.15, chi-squared test) or antecedent events for lower limb infection and/or gangrene (*P* = 0.058, chi-squared test) at the 5 % level of significance. Importantly, the samples did not differ by the main outcomes of any amputation (*P* = 0.068, chi-squared test) or major amputation (*P* = 0.85, chi-squared test). These findings, though not confirmatory, reinforced our impression that the unrecorded patient-episodes were MCAR and that the study sample was therefore representative of the total population of eligible cases admitted to hospital during the data collection period.

Table 1 lists variables recorded and their frequency distribution in the study sample. Table 2 displays the frequency distribution of events immediately preceding or precipitating infection as well as the risk of any amputation and major amputation (transmetatarsal or higher) by antecedent event. Trauma as a group (namely closed puncture wounds, lacerations, contusion/blunt trauma and burns) constituted the most common antecedent event for infection at 41 episodes (32 %, CI; 24.1–40.9 %). Closed puncture wounds were the most common precipitant within the trauma group at 25 episodes (19.5 %, CI; 13.1–27.5 %). The most common individual antecedent event was acute idiopathic soft tissue infection/acute ulcer (39/128, 30.5 %, CI; 22.6–39.2 %), defined as cases presenting with soft tissue infection/abscess with macroscopically unbroken skin (17/39, 43.6 %) or with a break in the skin existing for less than one month prior to presentation (acute ulceration) and not attributable to any specific, extraordinary traumatic or other event. In all cases of acute ulcers classified within this group, patients had to be sure that breakdown of the skin (acute ulceration) followed initial appearance of soft tissue swelling and signs of inflammation and infection and did not occur prior. Chronic neuropathic ulcer was the second leading individual antecedent at 30 episodes (23.4 %, CI; 16.4–31.7 %) followed by closed puncture wounds (above) and critical limb ischemia (CLI) accounting for 10 episodes (7.8 %, CI; 3.8–13.9 %). CLI is defined as end-stage peripheral arterial disease as manifested by gangrene and/or ischemic ulcers on the dorsum of the foot or tips of the toes and/or rest pain in the foot.

**Table 2:** Antecedent events for lower limb infection (and/or gangrene) and amputation

Antecedent/precipitating event	Frequency (%)	^a^No.(% of total) amputation	^b^No.(% of total) major amputation
Acute idiopathic soft tissue infection/ulcer^c^	39 (30.5 %)	26 (34.7 %)	11 (26.8 %)
Chronic neuropathic ulcer	30 (23.4 %)	18 (24 %)	9 (22 %)
Closed puncture wound	25 (19.5 %)	^d^6/23 (8 %)	^d^2/23 (4.9 %)
Critical ischemia	10 (7.8 %)	^e^9/9 (12 %)	^e^9/9 (22 %)
Bruise/blunt trauma	9 (7 %)	7 (9.3 %)	4 (9.8 %)
Laceration	5 (3.9 %)	3 (4 %)	1 (2.4 %)
Furuncle on lower limb	2 (1.6 %)	0	0
Chronic leg ulcer (venous)	2 (1.6 %)	1 (1.3 %)	1 (2.4 %)
Hot water burn	1 (0.8 %)	1 (1.3 %)	1 (2.4 %)
Sunburn	^f^1 (0.8 %)		
Ingrown toenail	1 (0.8 %)	1 (1.3 %	0
Toenail fungus	1 (0.8 %)	1 (1.3 %)	1 (2.4 %)
Bullosis diabeticorum	1 (0.8 %)	1 (1.3 %)	1 (2.4 %)
Re-infection of surgical wound	1 (0.8 %)	1 (1.3 %)	1 (2.4 %)
Total	128 (100 %)	75 (100 %)	41 (100 %)

^a^Amputation at any level out of 124 patient-episodes (four patient-episodes lost to follow-up)

^b^Major amputation, defined as transmetatarsal or higher, out of 124 patient-episodes (four patient-episodes lost to follow-up)

^c^This category includes soft tissue infections with unbroken skin or ulcers present for less than 1 month (in which temporal continuity between initial soft tissue infection and breakdown of the skin occurred over this period)

^d^Two patients, who did not have an amputation during initial admission, were subsequently lost to follow-up

^e^One patient with critical limb ischemia and gangrene who was offered amputation discharged himself from hospital and was lost to follow-up

^f^This patient developed superficial dry gangrene of her second toe and was offered amputation but refused and was lost to follow-up

Acute idiopathic soft tissue infection/ulcer accounted for the largest proportion of amputations (26/75, 34.7 %, CI; 24–46.5 %) followed by chronic neuropathic ulcer (18/75, 24 %, CI; 14.9–35.3 %), critical limb ischemia (9/75, 12 %, CI; 5.6–21.6 %), contusion/blunt trauma (7/75, 9.3 %, CI; 3.8–18.3 %) and closed puncture wound (6/75, 8 %, CI; 3–16.6 %). Acute idiopathic soft tissue infection also accounted for the largest proportion of major amputations (11/41, 26.8 %, CI; 14.2–43 %) followed by chronic neuropathic ulcer (9/41, 22 %, CI; 10.6–37.6 %), critical limb ischemia (9/41, 22 %, CI; 10.6–37.6 %), blunt trauma (4/41, 9.8 %, CI; 2.7–23.1 %) and closed puncture wound (2/41, 4.9 %, CI; 0.6–16.5 %). Trauma as a group accounted for 22.7 % (17/75) of amputations in general and 19.5 % (8/41) of major amputations.

Footwear trauma as an antecedent event is conspicuously absent from Table 2. Any such cases are likely to have been subsumed under the acute idiopathic soft tissue infection/ulcer category. The authors were reluctant to attribute any episode to footwear trauma *per se* unless there was a clear temporal connection, of which the patient was aware, between the wearing of uncomfortable shoes and appearance of the acute soft tissue infection/ulcer – no such cases were identified in this sample.

A nail lying in the yard was the implement most commonly causing closed puncture wounds (11/25, 44 %, CI; 24.4–65.1 %). Remaining puncture wounds were caused by a wide variety of implements, including shards of metal, glass and sharp rock. The predominant mechanism of blunt trauma was walking on or colliding with jutting stones or gravel (4/9, 44.4 %) usually whilst wearing flip flops or similar footwear.

The single case of hot water burn occurred when the patient soaked his feet in “warm” water and the patient with sunburn had the toes of her feet exposed to midday sun for a prolonged period of time at a sporting event.

Table 3 shows independent variables associated with the three most common antecedent events at a *P*-value of ≤ 0.1 after univariate testing, as well as changes in *P*-values following adjustment by multivariable logistic regression where appropriate. Presenting with acute idiopathic soft tissue infection/ulcer (versus other antecedents of lower limb infection and gangrene) was associated with male gender (*P* = 0.046) and non-ulcerative structural abnormalities of the foot (healed wounds from previous surgery, callus, bony or joint deformity, arch abnormalities) (*P* = 0.011), but not with any other variables, and both associations remained significant at the 5 % level after adjustment by multivariable logistic regression.

**Table 3:** Independent variables associated with the three most common antecedent events at a *P*-value of ≤0.1

Antecedent event	Associated variable	No. (%) or mean with antecedent event	No. (%) or mean with other antecedent events combined	OR; CI; *P*-value (*X* ^2^, Fisher’s exact or *t*-test)	Adjusted *P*-value (multivariable logistic regression)
^a^Acute idiopathic soft tissue infection/ulcer (*n* = 39)	Male gender	19/39 (48.7 %)	27/89 (30.3 %)	2.18; 1.01-4.73; 0.046	0.025
	^b^Non-ulcerative structural foot abnormalities	16/33 (48.5 %)	12/54 (22.2 %)	3.29; 1.29–8.14; 0.011	0.031
Chronic neuropathic ulcer (*n* = 30)	^c^Diabetes > 5 years	30/30 (100 %)	83/97 (85.6 %)	*P* = 0.027	
	^d^Previous infection affected limb	25/30 (83.3 %)	20/97 (20.6 %)	19.25; 6.54–57; <0.001	^d^ < 0.001
	^e^Previous infection either limb	29/30 (96.7 %)	36/97 (37.1 %)	49.14; 6.42–376; <0.001	^e^ < 0.001
	Insulin dependent	24/30 (80 %)	57/97 (58.8 %)	2.81; 1.05–7.49; 0.034	0.042
	^f^Peripheral sensory neuropathy	30/30 (100 %)	77/97 (79.4 %)	*P* = 0.007	
Closed puncture wound (*n* = 25)	^g^Previous infection affected limb	4/25 (16 %)	41/103 (39.8 %)	0.29; 0.09–0.9; 0.025	^g^0.007
	^h^Previous infection either limb	6/25 (24 %)	59/103 (57.3 %)	0.24; 0.087–0.64 0.003	^h^0.001
	Insulin dependent	10/25 (40 %)	72/103 (69.9 %)	0.29; 0.12–0.71; 0.005	0.003
	Time to first presentation to a doctor	4.7 days	7.6 days	0.9; 0.81–1.005; 0.056	0.015

^a^All idiopathic soft-tissue infection/acute ulcers occurred in weight bearing areas of the foot, 35/39 (89.7 %) occurring on the sole of the forefoot or toes, 3/39 (7.7 %) occurring on the lateral border of the instep and 1/39 (2.6 %) occurring on the heel. Six cases were associated with underlying osteomyelitis

^b^Non-ulcerative structural abnormalities included any that distorted the normal contour of the foot (bony or joint abnormalities, callus, etc.)

^c^All patients with chronic neuropathic ulcer as antecedent event had been diagnosed with diabetes for more than 5 years. STATA could not calculate an OR and dropped this variable from the multivariable logistic regression output

^d,e^These two variables were alternated in the logistic regression equation because of the high degree of correlation between them. The *P*-values from the logistic regression output therefore reflect the effect of either variable but not both at the same time

^f^All patients with chronic neuropathic ulcer had peripheral sensory neuropathy. STATA could not calculate an OR and dropped this variable from the multivariable logistic regression output

^g,h^These two variables were alternated in the logistic regression equation because of the high degree of correlation between them. The *P*-values from the logistic regression output therefore reflect the effect of either variable but not both at the same time

Chronic neuropathic ulcers causing infection had existed for 35 days to 6 years (mean 1 year, 8 months) prior to onset of infection. The plantar aspect of the forefoot and toes was the predominant site of neuropathic ulcers (66.7 %) with 26.7 and 6.7 % occurring on the heel and lateral margin of the instep respectively. In 14/30 (46.7 %) cases, the appearance of the chronic ulcer was preceded by an event with spontaneous occurrence in the remainder. Five chronic ulcers followed blunt trauma or contusion, five closed puncture wounds, two lacerations and two followed surgical debridement for soft tissue infection. Having chronic neuropathic ulcer as a precipitant of infection was strongly associated with being diagnosed with diabetes at least 5 years (*P* = 0.027), previous infection in the affected limb (*P* < 0.001), previous infection in either limb (*P* < 0.001), insulin dependence (*P* = 0.034) and peripheral neuropathy (*P* = 0.007) (Table 2).

Closed puncture was the only precipitant occurring within the trauma group in a sufficient number to permit statistical analysis. The odds of presenting with a puncture wound was significantly less among patients with one or more previous infections in the affected limb (*P* = 0.032), one or more previous infections in either limb (*P* = 0.004) and insulin-dependent diabetics (*P* = 0.007). Time to first presentation to a doctor after recognition of the antecedent event was less in patients with puncture wounds (4.7 days, CI: 2.9–6.6 days) than for other causes combined (7.6 days, CI: 6.2–9 days) and the difference, of borderline significance on univariate testing (*P* = 0.056), became highly significant (*P* = 0.015) when all three variables were included in logistic regression equations (Table 2), with one or more previous infection in the affected limb and infection in either limb being tested in separate equations.

Critical limb ischemia (CLI) was the antecedent event in only 10 episodes, a number not permitting of robust statistical analysis, hence its exclusion from Table 2. Nevertheless, some cogent observations are warranted. The age in years of patients with CLI was higher (mean 70.4, range 64.3–76.5) than that of those with other antecedents of infection combined (mean 57.4, range 54.9–60) (*P* = 0.0038, *t*-test). Eight were female and two male. Five (all women) had never smoked and three (two women and one man) were current smokers. All 10 had been diagnosed with diabetes for more than five years (five more than 20 years) and all were hypertensive. None had palpable distal pulses (dorsalis pedis or posterior tibial) and all had ankle-brachial index (ABI) ≤ 0.4 and dry or infected gangrene (limbs with ABI ≤ 0.4 but without clinical signs of critical ischemia were not included in this group). Eight had below-knee (BK) amputations and one above-knee (AK) – the remaining patient was offered AK amputation but refused and was subsequently lost to follow-up.

## Discussion

The antecedents of serious lower limb infection (requiring hospitalization) and gangrene identified in this representative sample of adult diabetics in Jamaica are, in order of decreasing frequency, acute idiopathic soft tissue infection/ulcer (39/128, 30.47 %, CI; 22.64–39.22 %), chronic neuropathic ulcer (30/128, 23.44 %, CI; 16.41–31.74 %), closed puncture wounds (25/128, 19.53 %, CI; 13.06–27.47 %) and critical limb ischemia (10/128, 7.81 %, CI; 3.81–13.9 %) with miscellaneous events (including other mechanisms of trauma) accounting for the remainder. As a group, trauma (specifically defined as closed puncture wounds, lacerations, contusions/blunt trauma and burns) constituted the most common antecedent event at 41 episodes (32.03 %, CI; 24.06–40.85 %).

Antecedents of amputation at any level are, in order of decreasing frequency, acute idiopathic soft tissue infection/ulcer (26/75, 34.7 %, CI; 24–46.5 %), chronic neuropathic ulcer (18/75, 24 %, CI; 14.9–35.3 %), critical limb ischemia (9/75, 12 %, CI; 5.6–21.6 %), contusion/blunt trauma (7/75, 9.3 %, CI; 3.8–18.3 %) and closed puncture wounds (6/75, 8 %, CI; 3–16.6 %).

These findings confirm our suspicion that chronic neuropathic ulceration is not the most common antecedent of either diabetic foot infections or amputation in this population.

One of the reasons making it difficult to interpret and compare studies on antecedent factors for lower limb infection in diabetics is that no two studies use similar classifications for such events or even the same definition of commonly used terms such as trauma, infection, diabetic foot, foot lesions and ulcer. For example, Islam et al. [19], in a prospective study of 446 patients admitted to hospital with diabetic foot infections, identified trauma (50.7 %), footwear related injuries (42.4 %), lacerations (2.2 %) and burns (1.6 %) as the causes of infection. No cases of infection seem to have been attributed to chronic neuropathic ulcer, which is unlikely, or else this cause was included under the umbrella categories of “trauma” and “footwear related injuries”. Trauma was not defined (and apparently excluded footwear related injuries, lacerations and burns which were described separately) and the authors did not indicate the methodology by which they identified footwear related injury as the cause of a given infection. Aziz et al. [20] identified types of diabetic foot infection in their study as abscess (32 %), wet gangrene (29 %), infected ulcers (19 %), osteomyelitis (13 %), necrotizing fasciitis (4 %) and cellulitis (3 %). Except for those infections caused by “ulcers”, there is no indication of the antecedents of any of the other types of infection. Infection itself is also not clearly defined in some publications, leading to the possibility of confusing cases of mere contamination with active infection, afflictions which evoke different therapeutic strategies and urgency. Most confusing is the widespread use of the term “ulcer” without any qualification to indicate whether reference is being made to chronic neuropathic ulcer, acute ulcer or breaks in the skin from any cause (including skin breaks caused by penetrating or lacerating injury and surgical debridement).

We identified no other publication that took the approach used in this study. We felt that it was important to identify precipitating causes where an unequivocal, temporally consistent, plausible connection existed between the occurrence of an event or presence of a pre-existing condition, such as a closed puncture wound or chronic neuropathic ulcer, and the occurrence of limb threatening infection. On the other hand, we felt that it was sufficient to describe the event or condition preceding limb threatening infection when no causative factor could be unequivocally identified, such as with acute soft tissue infection/ulceration. Hence the use of “antecedent” rather than cause to describe both these types of events and conditions.

There are surprisingly few studies prospectively researching the antecedents of lower limb infection in diabetics, an observation also made by others [21]. Since most if not all lower limb amputations in diabetics are performed because of such infections, knowledge of the antecedents of infection is therefore critical if amputation reduction strategies are to be evidence-based.

Although identifying the antecedents of infection was not the primary aim of the study by Lavery et al. [21], they reported that all but one of 151 patients who developed a foot infection had a pre-existing wound or penetrating injury, with wound being broadly defined as a “full skin thickness lesion involving any portion of the foot or ankle”. In the legend of an accompanying table the authors state that “Of 151 ft infections.....all but 1 (150) involved a penetrating wound or ulcer”, implying that all wounds that were not due to penetrating injury were in fact ulcers. Of the wounds associated with infection, 96.7 % had been present for more than 30 days, meaning that only 3.3 % (one) would have had an acute lesion as defined in our study.

Aziz et al. [20] reported abscess as the type of infection in 32 % of their cases. Since they report infections associated with ulcers separately at 19 %, it is presumed that abscesses represent the type of idiopathic acute soft tissue infection referred to in our study. Some acute idiopathic soft tissue infections may also have been subsumed in the wet gangrene (29 %), necrotizing fasciitis (4 %) and cellulitis (3 %) categories of that study [20].

Tobalem and Uckay [22] give an excellent description of the kind of lesion we have described as idiopathic acute soft tissue infection/ulcer, the only difference being that the cases in our study all occurred on weight bearing parts of the sole rather than on the dorsum of the foot as in their case report. An early stage of this idiopathic acute soft tissue infection/ulcer lesion seems to have been recognised in the University of Texas diabetic wound classification system [23] as a 0-B wound (pre-ulcerative lesion with cellulitis) but not at all in the Wagner classification [23]. The IDSA and International Working Group on the Diabetic Foot (IWGDF) classifications of diabetic foot infections do not speak to the presence or absence of overlying ulceration [14].

Why is this lesion not reported more commonly as an antecedent or class of foot infection? It could be that acute idiopathic soft tissue infection/ulcer is being camouflaged by being grouped under a putative causative factor, such as footwear injury or “trauma”, rather than being reported as an entity in itself. Acute idiopathic soft tissue infection/ulcer more likely results from the cumulative effect of repetitive and sustained stress to the feet from multiple causes, including walking, standing and other ordinary trauma of everyday life, culminating in microtrauma to the skin as an avenue for entry of bacteria. Asumanu et al. [24] wisely attributed no cause or aetiology to 80 % of soft tissue infections and abscesses in diabetics in their study (though not all cases affected the lower limbs). Another plausible explanation for the underreporting of this lesion is that those acute lesions in which the skin has broken down are being lumped with ulcers in general, as are those with intact skin subjected to surgical debridement (iatrogenic ulceration).

Of concern is that if acute idiopathic soft tissue infection/ulcer continues to be reported as caused by specific aetiologies, without much evidence to support such assumptions, rather than as a specific lesion, it will take longer for us to adequately understand its pathogenesis. Such knowledge will be critical to planning and implementation of effective interventions to prevent and treat it at its earliest stage. Lavery et al. [21] expressed surprise that the incidence of foot infection was so high in a cohort of patients who had been “extensively educated, provided with therapeutic shoes…, followed in a foot clinic and having ready access to podiatric care”; in other words, despite being offered the highest possible level of prevention and care that our current state of knowledge allows. Implicit in this expression of surprise is an admission that enough is not yet known about the pathogenesis of foot infections in diabetics to enable more effective prevention.

It is also possible that this lesion is genuinely more common as an antecedent of serious lower limb infection (at 30.47 %) in our diabetic population than it appears to be in the population studied by Lavery et al. [21] (at 3.3 %) in Texas. Bharara et al. [25] describe a phase of soft tissue inflammation which appears to presage breakdown of the skin and chronic neuropathic ulceration, possibly after becoming secondarily infected and presenting as the pre-ulcerative lesion recognised in the University of Texas classification system [23]. It is plausible that this same initially inflammatory lesion, when secondarily infected via microtraumatized skin, is what evolves into the acute soft tissue infection/ulceration described herein. If indeed this inflammatory lesion represents a common pathway why does it apparently progress to indolent, chronic, neuropathic ulceration in the population studied by Lavery et al. [21] rather than full blown, limb threatening infection as seems to be the case in our Jamaican sample? This could, at least in part, be due to the very high levels of poor glucose control in the Jamaican diabetic [26].

Variables identified in Table 3 as being statistically associated with the antecedent or precipitating factors for foot infection and gangrene after multivariable regression may be helpful in alerting physicians to the imminence of their occurrence. For example, men with any non-ulcerative abnormality of the contour of the foot (whether due to bony or joint deformity, raised skin lesions, arch deformities, etc.) are predisposed to occurrence of acute soft tissue infection/ulceration. Chronic neuropathic ulcers are more likely to develop in insulin-dependent diabetics diagnosed more than 5 years earlier and who also manifest peripheral neuropathy. A history of prior lower limb infection, more common in patients with chronic neuropathic ulcers, may well reflect previous episodes due to the ulcer rather than predisposing to its occurrence. Variables associated with the odds of presenting with a closed puncture wound are unhelpful and merely suggest that patients with prior lower limb infections and insulin dependent-diabetes tend to walk about less and are therefore less susceptible to accidental injury. It is not surprising that patients sustaining a closed puncture wound present earlier for medical care than those with other antecedents (4.7 versus 7.2 days) given that a puncture wound is a clear-cut, identifiable event more likely to demand patient attention and action than those antecedents which are of more subtle onset. Variables associated with critical ischemia are already well known. In this study, smoking as a risk factor for peripheral arterial disease seems to be receding into the background as diabetes and hypertension take center stage, although the small number in this group is not sufficient to enable any robust conclusions in this regard.

Management of advanced lower limb infections, with or without skin breakdown and regardless of the underlying cause, is settled knowledge; necrotic and infected tissue must be debrided to the interface with uninfected tissue. But questions remain regarding appropriate management strategies when patients present in the earliest stages of evolution of idiopathic soft tissue infections and closed puncture wounds, two antecedents identified as prevalent in this study. Specifically, what should be done for early soft tissue infections with unbroken skin (the 0-B pre-ulcerative wound of the University of Texas diabetic wound classification system) or the even earlier inflammatory lesion described by Bharara et al. [25]? Should these also be debrided with the attendant risk of precipitating a chronic ulcer in patients with insensate feet or is there a window during which offloading and antibiotic therapy might prevent worsening infection? In the case of closed puncture wounds presenting before infection sets in or soon after, would routine or selective deroofing reduce or increase the risk of severe infection and its consequences? This latter question was raised in a previous publication [27]. Further research is required to resolve these questions as they are of considerable importance in diabetic populations with high prevalence of these two types of foot infection. International guidelines should address these knowledge gaps.

The large number of missed patient-episodes thwarted the plan to recruit consecutive admissions and held the potential to introduce significant selection bias. However, patient-episodes appeared to have been missed completely at random. Moreover, some important data items that could be reasonably accurately identified retrospectively from the records of missed patient-episodes were compared with similar prospectively collected data from study participants and no statistically significant differences were identified. Any selection bias resulting from missed cases is therefore expected to be minimal.

Questions meant to unearth patients’ knowledge and behaviour with respect to foot care and injury avoidance were poorly constructed. Rather than asking if they were aware that diabetics require extraordinary attention to their feet and whether they paid any special attention to their feet, they should have been requested to explain exactly what they know and do. The variables constructed from these questions (“foot-care knowledge” and “foot-care behaviour”) are likely to have been inaccurate because of social desirability bias. This qualitative data will be important in gauging the need for and likely impact of the educational component of any amputation reduction strategy and should be the focus of additional research.

## Conclusions

Chronic neuropathic ulcer accounted for only 23.4 % of lower limb infections and 27.7 % of amputations in this population of diabetics, making it the second most common antecedent of either after acute idiopathic soft tissue infection/ulcer at 30.5 and 34.7 % respectively. Trauma as a group (defined as closed puncture wounds, lacerations, contusions/blunt trauma and burns) also accounted for a greater number of lower limb infections but fewer amputations than chronic neuropathic ulcer, at 32 and 19.5 % respectively.

Variables statistically associated with non-traumatic antecedents may be predictive of their imminence. Acute idiopathic soft tissue infection/ulcer was more likely to occur in men with any non-ulcerative foot deformity (defined as any deformity which distorts the normal contour of the foot, including joint, bony and arch abnormalities and calluses). Chronic neuropathic ulcer was more likely to occur in patients with diabetes for more than five years, insulin dependent diabetes and, as expected, peripheral sensory neuropathy.
